# Clinical pathological characteristics and treatment outcomes of renal cell carcinoma (RCC): a retrospective study from Sudan

**DOI:** 10.3332/ecancer.2023.1524

**Published:** 2023-03-23

**Authors:** Mudathir Mohamed Bafadni, Yassin Mohammed Osman, Mohammed El Imam Mohammed Ahmed, Mussab Mahjoub Taha, Dafalla Abu Idris, Khalid Eltahir Khalid Kheiralla, Moawia Mohammed Ali Elhassan, Mohamed Daffalla Awadalla Gismalla, Sami Mahjoub Taha Awad

**Affiliations:** 1 Department of Surgery, Faculty of Medicine, Al Zeem Al Azhari University, Khartoum 13311, Sudan; 2 Department of Urology, Gezira Hospital for Renal Disease and Surgery, Ministry of Health Medani, Medani 11111, Sudan; 3 Department of Surgery, Faculty of Medicine, University of Gezira, Medani 2667, Sudan; 4 Department of Oncology, National Cancer Institute, University of Gezira, Medani 2667, Sudan; 5 Department of Biochemistry and Nutrition, Faculty of Medicine, University of Gezira, Medani 2667, Sudan

**Keywords:** renal cell carcinoma, epidemiologic characteristics, disease free survival, sub-Saharan Africa, Sudan

## Abstract

**Background:**

Worldwide, renal cell carcinoma comprises 2.2% and 1.8% of global cancer incidence and mortality, respectively. Studies of epidemiology, treatment modalities and outcomes of renal cell carcinoma (RCC) in Sudan are scarce. To address this shortcoming, we evaluated baseline information on the epidemiology, types of treatment and outcomes of RCC at Gezira Hospital for Renal Diseases and Surgery (GHRDS) and the National Cancer Institute (NCI).

**Methods:**

We performed a retrospective, descriptive study of all patients with RCC, who were treated in GHRDS and NCI from January 2000 to December 2015.

**Results:**

A total of 189 patients with RCC were identiﬁed over the study period. Tumours were more common among male patients (56%) and involved the left kidney in 52% of cases. The median age at diagnosis was 57 years (range: 21–90 years). Loin pain was the most frequent symptom (*n* = 103 patients) followed by weight loss (*n* = 103 patients) and haematuria (*n* = 65 patients). The most common histopathologic type of RCC was clear cell (73.5%), followed by papillary (13.8%) and chromophobe (1.6%). The relative frequencies of stages I–IV were 3.2%, 14.3%, 29.1% and 53.4%, respectively. The overall median survival rate was 24 months, and the 5-year survival rate was 40%. The 5-year survival rate in stages I–IV was 95%, 83%, 39%, and 17%, respectively. Advanced stages and higher-grade tumour were associated with worse survival. The median survival of stage IV patients was better for patients who underwent nephrectomy (11.0 months) compared to those who did not undergo nephrectomy (4.0 months) (*p* value = 0.28).

**Conclusion:**

Our findings reveal poor outcomes for patients with RCC in Sudan, which is most likely due to the high proportion of patients presenting with advanced stages at the time of initial presentation.

## Introduction

Worldwide, renal cell carcinoma (RCC) is the 16th most common cancer, with 431,288 new cases of RCC and 179,368 deaths predicted in 2020, comprising 2.2% and 1.8% of global cancer incidence and mortality, respectively [1]. The incidence of RCC has had a significant rise globally. This can be partly explained by the increasing use of abdominal imaging and disease registration [2]. The incidence of RCC significantly differs among individual countries and world regions with the highest incidence in Western countries and lowest incidence in Africa [3]. Renal cell carcinoma occurs more commonly in males, with a peak incidence between 60 and 70 years of age [4]. Haematuria and palpable abdominal mass are rare (6%–10%) and correlate with aggressive histology and advanced disease [5]. Some patients present with symptoms caused by metastatic disease, such as bone pain or persistent cough [6]. The most common histological types have been reported to be clear cell carcinoma which represents 75%–80% of RCC, papillary (10%–15%) and chromophobe (5%). Other rarer forms such as collecting duct carcinoma (<1%) comprise the remainder [7]. Prognosis for patients with RCC is known to be related to the histological type, tumour, nodes and metastasis (TNM) classification and grade. The cancer-specific survival rate at 5 years after radical nephrectomy for patients with RCC is strongly associated with the histological types and tumour grades [8, 9].

In Sudan, data on clinical profile and treatment outcome of RCC is limited. Here, we provide baseline information about clinical characteristics, treatment and outcomes of renal cancer in a tertiary cancer referral centre in Sudan (as a low-income country in Africa).

## Methods and materials

### Study type

This is a retrospective, descriptive hospital-based study, involving all patients with histologically confirmed RCC treated at the Gezira Hospital for Renal Diseases and Surgery (GHRDS) and National Cancer Institute (NCI) between January 2005 and December 2015.

### Study area

Surgical operations were performed at the GHRDS, located in Wad Medani city, the capital of the Gezira state, which serves the whole Gezira state and the nearby states. The other institute that participated in the study was NCI; the only cancer treatment facility in the Gezira state. The cancer treatment modalities available at the NCI included radiotherapy (RT), systemic therapy (interferon alfa) and palliative care. GHRDS and NCI work in partnership through a multidisciplinary team meeting including an oncologist, urologist and pathologist; where all confirmed cases were meticulously discussed and clear road map of treatment made for each. Based on the aforementioned setting, cases with age ≥ 18 years diagnosed and histologically confirmed RCC were included, while renal masses diagnosed as benign were excluded.

### Data collections

Data extracted from hospitals registry included demographics, clinical presentation, pathological reports (histologic subtypes and tumour grades), radiology reports, treatment modalities and survival outcomes. The clinical stages were classified according to the 6^th^ edition of the staging system adopted by the American Joint Committee on Cancer TNM [10]. The histological subtypes (and grades) were determined at the University of Gezira Medical Laboratory, and were classified based on the World Health Organization classification for renal tumours [11]. The study protocol was approved by the Ethics Committee of the University of Gezira (UGEC) and the directors of each Centre.

### Statistical analysis

The data were coded, entered and analysed by the Statistical Package for the Social Sciences version 24 (SPSS Inc., Chicago, IL, USA). We analysed the descriptive data as frequencies and percentages for categorical variables or as both mean ± SD and median (range) for continuous variables. Survival analysis was performed using the Kaplan–Meier method. The association between tumour stages and survival was analysed using the log-rank test. Statistical significance was considered at p value of 0.05.

## Results

A total of 189 patients were included in this study. The mean and median ages at diagnosis were 58 years (SD, 13) and 57 years (range: 21–90 years), respectively. The incidence rate was highest between 50 and 60 years of age. Most patients were male (56%). Patient characteristics, including demographics, tumour locations and types of treatment, are shown in Table 1.

The majority (98.4%) of the patients were symptomatic, with only three patients detected incidentally. Loin pain was the most frequently reported clinical presentation (88%), followed by weight loss (55%), haematuria (34%), abdominal swelling (20%) and pathological fracture (2%). Forty-five (24%) patients had a single presenting symptom, 86 (46%) had two symptoms and 58 (30%) had three or more symptoms.

The American Joint Committee on Cancer (AJCC) staging of our study population was shown in Table 1. At presentation, the majority (54%) of patients had stage IV disease. Of the 101 patients with stage IV, the TNM staging was T4 any T and M0 in 26 (25.7%) cases, any T any N any M1 in 75 (74.3%) cases. Lung was the most common site of distant metastasis (37%), followed closely by bone (35%) then liver (20%) and other rare sites (8%).

Nephrectomy was performed in 66% of our study population (Table 1). Eleven (5.6%) patients with metastatic disease underwent nephrectomy either for pain, or in the remote hope of a spontaneous regression. RT was performed in 40% of the study cases, while systemic therapy in the form of interferon alfa was used to treat 22 (12%) patients. No patient received targeted therapy or immunotherapy.

At the last follow-up, 85 (45.0%) patients were alive, 96 (50.8%) patients were dead and survival status was unknown in 7 (4.2%) patients due to loss of follow-up. Most (85.4%) of the patients with grade 1 tumour were alive compared to 59.3% and 25.6% of the patients with grade II tumour and grade III tumour, respectively (*p* value < 0.001) as shown in Table 2. All (100.0%) stage I, almost all (96.4%) stage II, more than two-third of stage III and approximately 5% of stage IV patients were alive at last follow-up (*p* value < 0.001).

**Figure 1.**
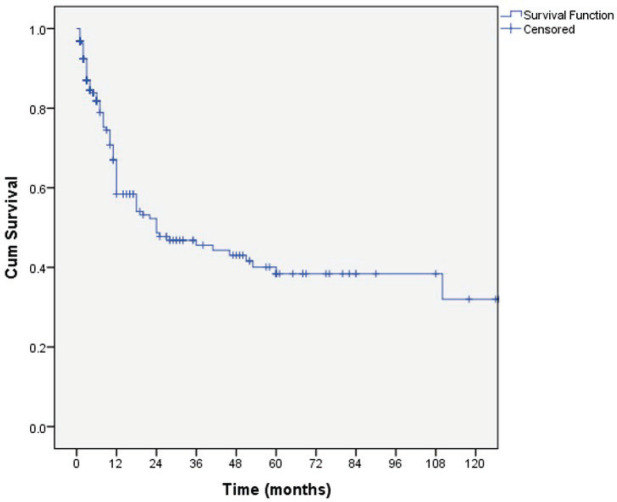
Kaplan–Meier curve of overall survivals.

**Figure 2.**
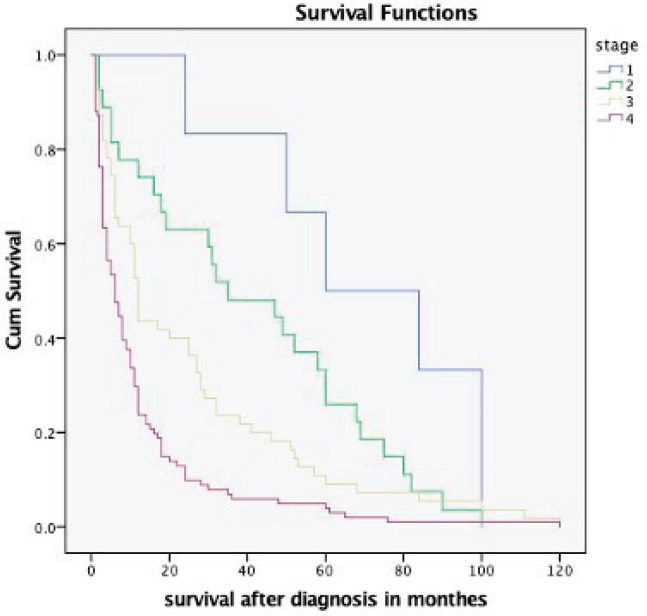
Kaplan–Meier survival curve depicting the survival of RCC patients based on different stages at presentation.

**Figure 3.**
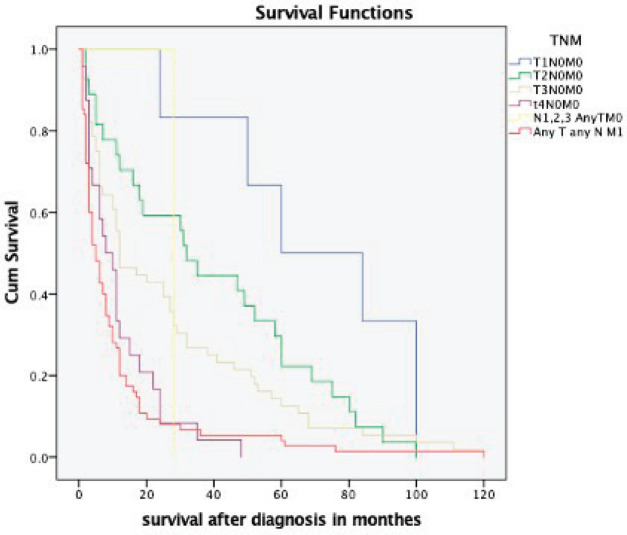
Kaplan–Meier survival curve depicting the survival of RCC patients based on different TNM stages.

The overall median survival rate was 24 months and the overall 5-year survival rate was 40% (Figure 1). The 5-years’ survival rate of stages I–IV was 95%, 83%, 39% and 17%, respectively (Figure 2). Furthermore the survival rate was related to the tumour size (Figure 3). A significant association between survival and clinical stage (*p* value <0.001) and tumour grade (*p* value = <0.001) of the disease was shown in Table 2. The median survivals of stage IV patients who underwent nephrectomy compared to those who did not undergo nephrectomy were 11.0 versus 4.0 months, respectively (*p* value = 0.28) (Table 3).

## Discussion

Kidney cancer accounts for 2.2% of total cancer burden globally [1]. Its incidence is higher in more developed countries than in less developed countries [12]. In Sudan, due to the lack of a population-based cancer registry, the main sources of data on cancer epidemiology are found in hospital case series. Awadelkarim *et al* [13] showed that the relative frequency of renal cancers reported in adult Sudanese patients, obtained by pooling data from published sources between 1959 and 2007 was 0.8%. The proportion of kidney cancers in this study seems to be low compared to that of other cancers. Possible reasons for this difference could be that the data were from a hospital and therefore are not a true representation of the cancer burden. Furthermore, underreporting may contribute to a low incidence of renal cancers in our resource-limited settings.

The estimated incidence of RCC increases in the older population, and incidence rates are significantly higher in males than in females across all age groups [3]. In this study, males were affected more than females and the peak incidence is between 50 and 60 years. Worldwide, the peak incidence of RCC is between 60 and 70 years [3]. The incidence rates of RCC have been increasing, in higher-income settings, this may be partially be due to an increase in the incidental detection of renal masses when abdominal imaging is performed for nonspecific gastrointestinal complaints. Previous studies conducted in the USA, China, and Spain showed that RCC was incidentally discovered in 60%, 25% and 6%, respectively. In this study, the majority of patients presented with locally advanced or metastatic disease and only 3 (1.2%) patients were discovered by the radiologists during scanning for other symptoms. Among our patients, loin pain, frank haematuria, weight loss and loin mass were the commonest features at presentation which commonly denote advance stage. This pattern is similar to that described in other low-income countries [3].

Radical nephrectomy, which has been recognised as the treatment of choice in localised RCC, was successfully performed in two-thirds of our study population. This finding was consistent with a previous study from Nigeria [3]. We noticed that, the mean time between diagnosis and intervention was 8.45 days. Early intervention eliminates the possibility that undue delay may have affected the stage and the prognosis. Clear cell carcinoma was the most common (73.5%) histological types in our study, in line with histological distribution worldwide [7]. In our limited-resource setting, more modern treatment options such as nephron-sparing and laparoscopic surgery are not available due to lack of training and inadequate infrastructural support.

This study showed that the majority of Sudanese patients with RCC presented with locally advanced or metastatic disease. This contrasts with high-income countries where most RCCs were discovered incidentally at an early stage [5, 15]. This high frequency of late stages at presentation in our institution may be a reflection of the limited diagnostic and treatment resources outside in the rural areas in Sudan and patients have to travel long distances to the referral centres in the country.

The 5-year survival rate among patients with RCC in the United States was increased from 57% in 1987−1989 to 74% in 2006−2012; this increase was attributable in part to a higher proportion of detection of asymptomatic disease at early-stage and indolent tumours identified by using abdominal images [5, 16]. Furthermore, new treatment options, including antiangiogenic drugs targeting Vascular Endothelial Growth Factor and its receptors, Mechanistic Target of Rapamycin inhibitors and an immune checkpoint inhibitor, have improved prognosis in patients with metastatic RCC [17]. The results of the present study demonstrate that the 5-year survival rate was 40%. This poor outcome is more likely due to late presentation and lack of targeted therapies in our resource-limited settings. We found 5-year survival rates of 95% for stage I and 83% for stage II RCC, decreasing to 39% among patients with stage III and 17% among patients with stage IV.

Flanigan *et al* [18] evaluated the overall survival of 241 advanced RCC patients, they were randomised to either nephrectomy followed by interferon or interferon alone the result was the median survival was 11.1 versus 8.1 months, respectively, favouring surgery plus interferon. In this study, the median survival of stage IV patients who underwent nephrectomy and those who did not undergo nephrectomy was 11.0 and 4.0 months, respectively. Our data showed a better survival in stage IV patients with nephrectomy, although a statistical significance was not reached. Targeted therapy and immunotherapy, the current standard of care for the management of stage IV RCC, are not available in Sudan due to their high cost. Therefore, in our resource-limited settings, nephrectomy could offer the best survival outcome for patients with stage IV RCC.

In this study, we were able to obtain records of a fairly large sample of cases that spanned 7 years, which was highly representative of RCC in our institute. However, the current study has limitations including its retrospective nature and reliance on medical records. Moreover, it is a single institution’s data; therefore, this data cannot be extrapolated for all Sudan. However, the NCI and GHRDS are the only cancer referral oncology institutes in central Sudan; therefore, the current data is the best indicator of the treatment outcomes of RCC within those centres.

## Conclusion

Our findings reveal poor survival outcomes for patients with renal cancer in Sudan, most likely due to a high proportion of patients who are diagnosed with stage IV disease at initial presentation and no access to targeted therapies or immunotherapy. More studies are needed to identify the reasons for presentation at advanced stages in our limited-resource settings.

## Conflicts of interest

The authors declare that they have no competing interests.

## Funding

This study did not receive a specific grant from any funding agency in the public, commercial or non-profit sectors.

## Figures and Tables

**Table 1.** Patients demographic and clinical characteristics.

Characteristics		Frequency (%)
Gender	Male	106 (56)
	Female	83 (44)
Age/years	20–29	2 (1)
	30–40	11 (6)
	40–49	29 (15)
	50–59	59 (31)
	60–69	48 (26)
	>70	29 (21)
Symptoms at diagnosis	Loin pain	106 (87.8)
	Weight loss	103 (54.5)
	Haematuria	65 (34.4)
	Abdominal swelling	37 (19.6)
	Fever	24 (12.7)
Tumour site (laterality)	Right	86 (45)
	Left	99 (52)
	Bilateral	3 (1.6)
Type of surgical intervention	Radical nephrectomy	125 (66.7)
	Partial nephrectomy	1 (0.5)
	Biopsy	51 (27)
	No intervention	12 (6.3)
RT	Adjuvant	44 (23)
	Palliative	32 (17)
	None	113 (60)
Systemic therapy	Interferon alfa	￼22 (12)
	Targeted therapy	0
	None	167 (88)
Stages	1	6 (3.2)
	2	27 (14.3)
	3	55 (29.1)
	4	101 (53.4)
Grade^a^	1	13 (6.9)
	2	86 (45.5)
	3	90 (47.6)
Type of cancer	Clear cancer	139 (73.5)
	Papillary	26 (13.8)
	Chromophobe	3 (1.6)
	Others	21 (7.4)

a Grades were only 3 points scale

**Table 2.** Association of survival outcome at last follow-up with grade and stage of renal cell carcinoma.

		Outcome				Total	*p* value
		Alive	Morbidity^a^	Disappear^b^	Died		
		(*n* = 65)	(*n* = 8)	(*n* = 7)	(*n* = 96)	(*n* = 189)	
Grade	Grade 1	9	2	0	2	13	
	Grade 2	38	13	1	34	86	<0.001
	Grade 3	18	5	6	61	90	
Stage	Stage 1	6	0	0	0	6	
	Stage 2	26	0	0	1	27	<0.001
	Stage 3	22	16	1	16	55	
	Stage 4	11	4	6	80	101	

a Alive with recurrence of the disease locally or distance

b Disappear from follow-up with no contact

**Table 3.** Relation of renal cell carcinoma stage with treatment.

		Stage				Total	*p* value
		Stage 1	Stage 2	Stage 3	Stage 4		
Adjuvant radiotherapy	Yes	0	7	16	21	44	0.36
	No	6	20	39	80	145	
Systemic therapy	Yes	0	1	5	16	22	0.01^a^
	No	6	26	50	85	167	
Surgical procedure types	Radical nephrectomy	5	27	45	48	125	0.001^a^
	Partial nephrectomy	1	0	0	0	1	
	Biopsy	0	0	9	42	51	
	No surgery	0	0	1	11	12	

a Correlation is significant at the 0.05 level
